# Clarifying Eudaimonia and Psychological Functioning to Complement Evaluative and Experiential Well-Being: Why Basic Psychological Needs Should Be Measured in National Accounts of Well-Being

**DOI:** 10.1177/17456916221141099

**Published:** 2023-01-10

**Authors:** Frank Martela, Richard M. Ryan

**Affiliations:** 1 Department of Industrial Engineering and Management, Aalto University; 2 Institute for Positive Psychology and Education, Australian Catholic University; 3 College of Education, Ewha Womans University

**Keywords:** national indicators of well-being, subjective well-being, eudaimonia, eudaimonic well-being, psychological functioning, psychological needs, self-determination theory

## Abstract

Measuring subjective well-being as a key indicator of national wellness has increasingly become part of the international agenda. Current recommendations for measuring well-being at a national level propose three separate dimensions: evaluative well-being, experiential well-being, and eudaimonia. Whereas the measurement of the first two dimensions is relatively standardized, the third category has remained undertheorized, lacking consensus on how to define and operationalize it. To remedy the situation, we propose that the third dimension should focus on psychological functioning and the identification of key psychological factors humans generally need to live well. A key part of psychological functioning is the satisfaction of basic psychological needs—specific types of satisfying experiences that are essential for psychological health and well-being. Psychological needs as a category provides a parsimonious set of elements with clear inclusion criteria that are strongly anchored in theory and our current understanding of human nature—and could thus form a core part of the third, “eudaimonic” dimension of well-being. The needs for autonomy, competence, and relatedness have especially received broad empirical support. Accordingly, national accounts of well-being should include measures for key psychological needs to gain an enriched and practically useful understanding of the well-being of the citizens.

Measuring subjective well-being as a way of evaluating the wellness and progress of nations has increasingly become part of the international agenda, from the United Nations (UN) General Assembly inviting member states “to pursue the elaboration of additional measures” (UN Resolution 65/309, 2011, p. 1) that assess happiness and well-being to the Organisation for Economic Co-operation and Development (OECD) recommending that all of its member countries start measuring subjective indicators of well-being (OECD, 2013). Subjective well-being refers to all subjective dimensions of human well-being, and the current “best practice for well-being questionnaires” on a national level includes measures for three separate dimensions (Graham et al., 2018,p. 287): evaluative well-being (life satisfaction), experiential well-being (positive and negative affect), and a third dimension alternatively called eudaimonia, eudaimonic well-being (EWB), or psychological functioning. We find this trichotomy in the recommendations of the U.S. National Research Council (2013), in the OECD guidelines on measuring subjective well-being (OECD, 2013), in the recommendations of the European Union’s expert group on quality-of-life indicators (Eurostat, 2017), as well as in the recommendations published in prominent journals such as *Science* (Graham et al., 2018) and *The Lancet* (Steptoe et al., 2015). Accordingly, the trichotomy has been implemented by the UK’s Office for National Statistics (ONS) in their Measuring National Well-Being Programme (ONS, 2018), by Eurofound’s (2017) quality-of-life surveys for all member states of the European Union, and by national initiatives in countries ranging from Mexico and Canada to New Zealand and South Korea (Stone & Krueger, 2018), whereas the Gallup World Poll has been used to examine evaluative, experiential, and eudaimonic well-being in more than 160 countries across the world (Joshanloo, 2018; Joshanloo & Jovanović, 2021). Currently, this trichotomy of evaluative well-being, experiential well-being, and eudaimonic/functional well-being thus quite prominently defines how the subjective dimensions of citizen well-being are measured around the world.

Although there is relatively much agreement over how to best define and operationalize evaluative and experiential well-being, the eudaimonic/functional well-being category has remained undertheorized. Although the OECD guidelines recommended its inclusion, they noted that “the conceptual structure of eudaimonic well-being is less fleshed out” (OECD, 2013, p. 32), a concern echoed also by the National Research Council (2013). Although experts thus agree that current measures for evaluative and experiential well-being are too narrow, missing out on important dimensions of well-being, we have yet to find a consensus on what those missing dimensions are. In fact, Martela and Sheldon (2019) identified at least 45 different ways of conceptualizing eudaimonic well-being, with many commentators complaining about the “looseness,” “vagueness,” and “lack of unification” around how to define and operationalize eudaimonia, eudaimonic well-being, and psychological functioning (e.g.,Heintzelman, 2018; Huta & Waterman, 2014; Kashdan et al., 2008). This is a major obstacle for a comparable and cumulative science of eudaimonia/functioning (Martela & Ryan, 2021).

Given that how eudaimonia/functioning is conceptualized directly influences how international organizations and governments around the world measure well-being—and thus what aspects of people’s well-being is visible to the policymakers—finding conceptual clarity and consensus is urgently needed in this area. Right now, such “broad consensus on best practice is still lacking” (Stone & Krueger, 2018, p. 169), leading to the usage of separate indexes with various ad hoc elements with little overlap, with frequent calls for more consensus around its core elements (Cooke et al., 2016; Heintzelman, 2018; Huta & Waterman, 2014; Martela & Sheldon, 2019; VanderWeele et al., 2020).

As a pathway toward such clarity and consensus, we see that an attempt to seek one right and final conceptualization for the ambiguous construct of eudaimonia might not be fruitful. Instead, the focus should be shifted to more clearly defined constructs, making independent judgments about whether they should be included in broader assessments of well-being. In particular, in this article we propose that one key category of the “third” dimension of well-being should be basic psychological needs. A focus on eudaimonic dimensions of well-being typically aims to identify key psychological factors humans generally need to do well in life, thus complementing the well-*being* focus of evaluative and experiential well-being with a focus on well-*doing* (Huppert et al., 2009; Martela & Sheldon, 2019; NEF, 2008; Ryan et al., 2008). Humans are biologically and psychologically constructed to universally require specifiable experiences to survive, thrive, and function well (Doyal & Gough, 1991; Pittman & Zeigler, 2007; Ryan & Deci, 2017). Human psychological functioning is thus crucially about the satisfaction of such universally present innate psychological needs, making psychological need satisfaction an integral and organic part of human well-being. Accordingly, psychological need satisfaction could provide one core focus for the various conceptualizations of psychological functioning and eudaimonic well-being by providing a parsimonious set of elements with clear inclusion criteria that are strongly anchored in theory and our current understanding of human nature (Martela & Sheldon, 2019).

Self-determination theory (SDT; Ryan & Deci, 2000, 2017), in particular, has made a strong empirical case for the existence of three basic psychological needs essential for human wellness, healthy psychological development, and optimal functioning: autonomy (sense of volition and self-direction), competence (sense of mastery, efficacy, and accomplishment), and relatedness (sense of mutually caring relationships; Deci & Ryan, 2000; Vansteenkiste et al., 2020). Given the large research evidence base supporting the importance of these three psychological needs for motivation, behavior, well-being, and health across the globe (Ng et al., 2012; Van den Broeck et al., 2016; Vansteenkiste et al., 2020; Yu et al., 2018), we propose that attempts to build broader assessments of well-being, especially for national accounts of well-being, should include measures for these basic psychological needs (Martela & Ryan, 2021; Martela & Sheldon, 2019). Although the empirical case for the needs for autonomy, competence, and relatedness is relatively strong, the exact nature and number of such needs is open for debate (see Martela & Ryan, 2020; Rasskazova et al., 2016; Ryan & Deci, 2017; Tay & Diener, 2011). Thus, our proposal about the inclusion of psychological need satisfaction into national accounts of well-being is not anchored to a specific set of needs but more generally proposes that the focus on psychological needs would provide clear criteria through which to empirically examine the case for the inclusion of any candidate element, resulting in a small and broadly agreed-on list of key elements. Such clear inclusion criteria could thus act as an antidote to the current development in which “an ever-expanding number of constructs become encompassed within an increasingly ambiguous” concept (Martela & Sheldon, 2019, p. 461). The focus on a small number of shared core elements would allow for the comparison of psychological functioning across countries and surveys, thus building a more cumulative science of well-being while providing for policymakers practically useful information about the well-being of the citizens and how to improve it.

We thus aim to contribute to research on national accounts of well-being by presenting an integrative review of how the third dimension of well-being has been conceptualized and operationalized. Specifically, we offer (a) our interpretation of a key dividing line within it between meaning/purpose and functioning/flourishing and (b) our proposal that the three psychological needs of SDT should form core components of the third dimension to be measured in future national and cross-national surveys.

## Evaluative, Experiential, and Functional Well-Being

Subjective indicators of well-being have increasingly established their place at a national level and in policy contexts as ways of measuring the progress, wellness, and sustainability of societies and nations in ways that go beyond the traditional economic metrics, such as the gross domestic product (GDP; National Research Council, 2013; OECD, 2013; Stiglitz et al., 2009, 2018). Advancing citizen well-being has always been an important policy goal—Adam Smith (1759/1982, p. 185) noted that all governments should be “valued only in proportion as they tend to promote the happiness of those who live under them.” However, although on an abstract level well-being of the citizens remained the goal of politics, in practice the era after World War II was dominated by GDP as the key indicator of national progress, closely followed by politicians, pundits, and the media (Coyle, 2014; Hoekstra, 2019). GDP, however, is known to overlook “externalities” such as the negative effects of traffic and pollution while including “regrettables” such as crime and worsening health, leading to more spending in these areas (Diener & Seligman, 2018). As the shortcomings and blind spots of GDP have become more apparent, politics began to seek alternatives to it that would better capture how the citizens and the nations are actually doing. Simultaneously, the psychological-research community started to more prominently advocate the benefits of national accounts of subjective well-being for policymakers (Diener, 2000; Diener et al., 2009; Diener & Seligman, 2004; Dolan & White, 2007). In 2007 the European Commission and OECD hosted the high-level conference “Beyond GDP,” followed by the influential report by Stiglitz et al. (2009) commissioned by French president Nicolas Sarkozy. In 2011, the UN General Assembly declared that “the pursuit of happiness is a fundamental human goal” and, given that “the gross domestic product indicator by nature was not designed to and does not adequately reflect the happiness and well-being of people,” it invited member states “to pursue the elaboration of additional measures that better capture the importance of the pursuit of happiness and well-being” (UN Resolution 65/309, 2011, p. 1). Various national and cross-national initiatives to measure subjective sense of well-being have ensued, leading to virtually all national statistics offices of OECD countries including subjective well-being questions into their annual surveys (Exton & Shinwell, 2018; Stone & Krueger, 2018).

One background reason for the rise of subjective well-being as a respectable policy instrument has been the advances in the science of measuring people’s perceptions of their own well-being that has taken place in the last decades (Diener, 2013; Diener et al., 1999; Dolan & White, 2007; Krueger & Stone, 2014). Research has demonstrated that, despite some acknowledged biases, subjective sense of well-being can be measured with enough precision to make nationally representative surveys useful policy instruments (Diener et al., 2018; OECD, 2013). Measures of subjective well-being have also been shown to predict various important factors from positive health outcomes (Howell et al., 2007) and longevity (Martín-María et al., 2017; Steptoe et al., 2015) to interpersonal and career success (Lyubomirsky et al., 2005) and how citizens within a country will vote in the next elections (Ward, 2020; Ward et al., 2021). Well-being measures have been also shown to track many hard-to-measure dimensions of quality of life not captured by economic indicators, such as clean air, hidden costs of living in noisy areas, access to nature, and social capital (Diener & Seligman, 2018; Dolan & White, 2007). Subjective sense of well-being is thus simultaneously an important and valued outcome in its own right, while providing “a summary of diverse dimensions of quality of life in society” (Diener, 2013, p. 665).

Although well-being is sometimes treated as a monolithic concept, the distinction between evaluative well-being referring to cognitive judgments and experiential well-being, referring to affective experiences, was acknowledged decades ago (Andrews & Withey, 1976; Diener, 1984; Lucas et al., 1996). Research has indeed shown that life satisfaction and positive affect are differently related to economic prosperity (Diener et al., 2010; Kahneman & Deaton, 2010; Kapteyn et al., 2015) and other factors such as unemployment (Knabe et al., 2010) and personality (Schimmack et al., 2008). *Evaluative well-being* refers to a reflective assessment of a person’s life as a whole (Diener et al., 1999; OECD, 2013). The measurement of such global judgment of one’s life has a long history with relatively standardized measures such as the Cantril ladder and various life-satisfaction measures that have been widely utilized in international surveys such as the World Values Survey. *Experiential well-being* refers to the everyday feelings, affects, and emotions experienced by the person. It thus aims to capture “peoples’ everyday emotions, their joys, miseries, and pains” (Stone et al., 2018, p. 360), with most experts recommending measuring positive and negative affects separately (Bradburn, 1969; Diener et al., 1999). The measurement of positive and negative affect has become relatively standardized, although the reliability of recalled emotions has been questioned, leading some to favor measuring emotions on a momentary basis or in short time frames (Kahneman et al., 2004).

However, several researchers have argued that, even together, affects and life satisfaction are not enough to cover all relevant dimensions of well-being (e.g., Clark et al., 2008; Huppert & So, 2013; Keyes, 2007), leading us to to “neglect important aspects of positive psychological functioning” (Ryff, 1989, p. 1070). They see that life is not only about a narrow hedonic focus on pleasures or satisfaction but also involves more eudaimonic dimensions of well-being such as personal fulfillment, fundamental need satisfaction, and realization of one’s potential. For example, research has shown that spending time with children is relatively more rewarding than pleasurable (M. P. White & Dolan, 2009), voting behavior is better predicted by anticipation about one’s future life evaluation rather than one’s current life evaluation (Ward et al., 2021), eudaimonic well-being is associated with lower levels of salivary cortisol and cardiovascular risk whereas positive affect is not (Ryff et al., 2004), and longevity is better predicted by purpose in life than life satisfaction (Martela et al., 2022). Human well-being is a broad, dynamic, and multidimensional phenomenon, the richness of which is not captured by just measuring life satisfaction and positive and negative affect (VanderWeele et al., 2020). To account for this multidimensionality of well-being and human flourishing, several researchers (Clark, 2016; Delle Fave, 2016; Dolan et al., 2011; Graham et al., 2018; Steptoe et al., 2015; Tennant et al., 2007) and expert groups (Eurostat, 2017; National Research Council, 2013; OECD, 2013) have suggested that experienced well-being should be examined using three separate dimensions: evaluative well-being (cognitive), experiential well-being (affective), and eudaimonic/functional well-being.

## The Third Dimension of Well-Being: Meaning in Life, Eudaimonia, or Psychological Functioning?

As seen, many parties recommend the inclusion of a third category into national accounts of subjective well-being. However, they simultaneously acknowledge that compared with evaluative well-being and experiential well-being with their fairly standardized assessments, this proposed third category is less clearly defined and accordingly lacks standardized and widely accepted measurements. The OECD noted that its conceptual structure is “less fleshed out” compared with the other two categories (OECD, 2013, p. 32), whereas the National Research Council (2013) noted that “its role in explaining behavior is less well understood” (p. 19). More recent reviews have also noted the ambiguity in how this third category of well-being is conceptualized and measured (Stone & Krueger, 2018), observing that “there is little agreement among scholars in this area regarding any one conceptual definition” (Heintzelman, 2018, p. 2). Observing that eudaimonic well-being has been defined or operationalized by constructs ranging from ego development and subjective vitality to flow and mindfulness, Martela and Sheldon (2019) concluded, “the vagueness of the EWB category seems to permit almost any operationalization at all,” which is “an untenable situation if the aim is to do comparable and cumulative science” (p. 461). The ambiguity starts already with the name for this category, which is variously referred to as eudaimonia (e.g., OECD, 2013; Stone & Krueger, 2018), eudaimonic well-being (e.g., National Research Council, 2013), psychological/positive functioning (Keyes, 2002), or flourishing (e.g., Huppert & So, 2013).

In general, there seems to be at least two partially overlapping but discernible ways of understanding this third category of well-being: seeing it as being about meaning and purpose or defining it in terms of psychological functioning. Regarding the first, the National Research Council (2013) defined eudaimonic well-being as referring to “people’s perceptions of the meaningfulness (or pointlessness), sense of purpose, and value of their life” (p. 19), whereas Graham et al. (2018) proposed that “eudaimonic metrics ask whether individuals have purpose or meaning in their lives” (p. 287; see also Steptoe et al., 2015). Eurostat (2017) also associated eudaimonia with “meaning and purpose of life” (p. 80). Both European Union’s Statistics on Income and Living Conditions and the UK’s ONS measure eudaimonia with one question: “Overall, to what extent do you feel the things you do in your life are worthwhile?” (Eurostat, 2017; ONS, 2018).

However, the second strong streak in the definitions of the third category of well-being is an emphasis on psychological functioning. For example, the OECD (2013) noted that eudaimonia focuses on “the concept of good psychological functioning” and is thus “concerned with capabilities as much as final outcomes” (p. 32). Tennant et al. (2007) saw that eudaimonic perspective is about “psychological functioning and self realisation” (p. 2), whereas Clark et al. (2008) observed that eudaimonia “captures functional aspects of well-being” (p. 122). Keyes, in turn, divided mental health into positive feelings and positive functioning, emphasizing that the latter also needs to be evaluated for a full understanding of mental health and human flourishing (Keyes, 2002, 2007). The European Social Survey’s well-being module similarly distinguishes between the hedonic approach concerned with pleasure, enjoyment, and satisfaction and the eudaimonic approach “concerned with functioning and the realisation of our potential” (Huppert et al., 2009, p. 5), a distinction echoed by the New Economics Foundation (NEF) in their recommendations on what to measure in national accounts of well-being (NEF, 2008). Whereas feeling and satisfaction relate to being well, positive functioning relates more to doing well (Huppert et al., 2009; NEF, 2008), thus enriching accounts of well-being with important information on how the person is doing and whether they are fully functioning (Ryan & Deci, 2001).

There are thus two alternatives that have been suggested to complement the traditional focus on life satisfaction and positive and negative affect: meaning/purpose in life and positive functioning. Sometimes this dualism of purpose and functioning is explicit in definitions of the third dimension of well-being, such as when Stiglitz et al. (2018) defined eudemonia as “the extent to which a person believes that his or her life has meaning and purpose, and is also related to a person’s psychological functioning” (p. 79). Similarly, Stone and Krueger (2018) first noted that eudaimonia is about whether a person’s life “has meaning and purpose” but a few sentences later noted that eudaimonia is used “to describe aspects of people’s psychological functioning” not covered by life evaluation or affects (pp. 165–166). While using meaning and purpose as a proxy for eudaimonia, Eurostat (2017) came to note that “eudemonic approaches to well-being aim to capture psychological functioning, the fulfilment of human potential, or a ‘life worth having’” (p. 83).

As regards this dualism between meaning and functioning in definitions of eudaimonia, the key conceptual point we want to make is as follows: Rather than competing definitions for eudaimonia, we should see them as two separate candidates for what to include in broader assessments of well-being. Just acknowledging this dualism is important. Instead of one unified third category of well-being beyond life satisfaction and positive and negative affect, there are two separate prime candidates for what such narrow focus is missing: judgments of the purpose and meaningfulness of one’s life and the psychological functioning present in that life. Both are important concerns in their own right for human life and well-being. Given the high degree of ambiguity around what eudaimonia is, the field might benefit by focusing on more clearly defined categories—such as psychological functioning and meaning in life. Thus, instead of seeing these two as competing for what eudaimonia truly is, we should treat them as two separate candidates for what to include in more comprehensive national accounts of well-being. Importantly, covering one does not cover the other. Including an indicator of purpose does not cover psychological functioning, and including indicators of psychological functioning do not cover meaning/purpose in life. Thus, whether meaning/purpose is included and whether psychological functioning is included in national accounts of well-being should be treated as two separate issues—with the present article focusing on the latter (we offer few suggestions about the role of meaning/purpose at the end of the article).

We see that psychological functioning could provide a good complement to experiential and evaluative well-being. Although evaluative and experiential well-being are about well-being in the sense of how life is generally felt, psychological functioning is about well-doing in the sense of focusing on how the subject is living their lives and whether certain key factors and experiences are present in their lives (Huppert et al., 2009; Martela & Sheldon, 2019; NEF, 2008; Ryan et al., 2008). Psychological functioning thus focuses on identifying the universally required psychological factors that humans need to do well in life and to feel well—psychological experiences deemed as central to human well-being, well-doing, and thriving. This focus on functioning is close to the roots of eudaimonia in Aristotelian philosophy, in which eudaimonia is seen to be more about activity and a way of living rather than a feeling (Annas, 1995; Aristotle, 2012; Ryan et al., 2013). Modern researchers following this conceptualization of eudaimonia see it as being about a “good and fulfilling way of life” rather than a specific experience (Ryan & Martela, 2016, p. 109) and thus focus on identifying the key motives, activities, and psychological needs that are good for humans and thus also typically lead to experiential and evaluative well-being (Huta & Ryan, 2010; Martela & Ryan, 2021; Martela & Sheldon, 2019). Identifying elements of psychological functioning is thus about identifying those specific psychosocial experiences that consistently and robustly lead to beneficial outcomes, such as well-being, personal growth, and integrity, as well as social adjustment and good life outcomes (Martela & Sheldon, 2019; Ryan et al., 2008).

One way of broadening well-being assessments would thus be to include indicators of psychological functioning alongside indicators of experiential and evaluative well-being. However, we also need clearer theory about what psychological functioning itself entails. Here, we see that psychological need satisfaction offers a good candidate for a central part of what psychological functioning should be about.

## The Satisfaction of Basic Psychological Needs as a Key Part of Psychological Functioning and Well-Being

Basic psychological needs refer to specific types of satisfying experiences a person can get from their interaction with the environment, postulated to be essential for the psychological health and well-being of the person (Deci & Ryan, 2000; Martela & Sheldon, 2019; Ryan & Deci, 2017). Postulating psychological needs thus proposes that there are “specifiable psychological and social nutrients which, when satisfied within the interpersonal and cultural contexts of an individual’s development, facilitate growth, integrity, and well-being” (Ryan & Deci, 2017, p. 82). Certain psychosocial experiences have proven so necessary for the survival and thriving of the organism that humans have developed robust psychological mechanisms that reward them emotionally when they are able to obtain these experiences, with a long line of research having aimed to identify such needs (Baumeister & Leary, 1995; Deci & Ryan, 1985; Maslow, 1943; reviewed in Pittman & Zeigler, 2007).

Given that psychological functioning is about doing well rather than just feeling well and thus draws from research aiming to identify what factors humans need to do and function well, universal psychological needs seem to fit very well within this conceptualization of functioning. Accordingly, conceptualizations of the third dimension of well-being emphasizing psychological functioning typically see that psychological needs play a key role in it. In defining eudaimonia, the OECD (2013) noted that it draws from traditions aiming to identify “key universal ‘needs’ or ‘goals’” (p. 32). Eurostat (2017) also mentioned “meeting psychological needs” (p. 83) in discussing the third dimension of well-being. Several theorists (e.g., Clark, 2016; Heintzelman, 2018; Kapteyn et al., 2015) have similarly emphasized that the third, eudaimonic dimension of well-being builds on theories conceiving “of us having underlying psychological needs” (Dolan et al., 2011, p. 9). Psychological needs as “universal, cross-developmental propensities upon which integrated functioning depends” (Ryan & Deci, 2017, p. 82) thus arguably form a key part of human psychological functioning. There could be other dimensions to psychological functioning, but the advantage of psychological needs is that the theory around them has provided clear criteria for what elements to include—and what not to include—when evaluating need satisfaction, thus providing a parsimonious set of indicators about central psychological dimensions of human functioning. Accordingly, psychological needs provide one core part of what assessments of psychological functioning, and more broadly the third dimension of well-being, should include.

Arguably among the most well-researched contemporary theories of basic psychological needs is SDT (Ryan & Deci, 2000, 2017), which identifies three psychological needs for autonomy, competence, and relatedness. *Autonomy* refers to feeling that one’s behavior is self-endorsed and volitional, *competence* refers to feeling effective and efficient in one’s actions, and *relatedness* refers to feeling connected to, and cared for, by important others. According to SDT, all three needs are independently important to well-being, and thus satisfaction of one need cannot fully compensate for the satisfaction of another (Sheldon & Niemiec, 2006). Moreover, evidence points to the relevance of these basic needs to wellness even in conditions of threat or insecurity (e.g., Lera & Abualkibash, 2022; Vermote et al., 2022). Although SDT has been the theory most focused on these three needs, it is worth noting that many other theoretical frameworks have also separately proposed relatedness (Alderfer, 1972; Baumeister & Leary, 1995; Maslow, 1954), competence or efficacy (Bandura, 1977; R. W. White, 1959), and autonomy (Doyal & Gough, 1991; Yu et al., 2018) as fundamental psychological needs essential to well-functioning.

The advantage of focusing on psychological needs as one core part of psychological functioning is that clear empirical inclusion criteria can be given for determining whether a certain proposed element should be seen as a psychological need. Instead of ad hoc listing of various elements, the selection of a parsimonious set of indicators for psychological functioning can thus be made on the basis of explicit criteria. Based on research within self-determination theory (Deci & Ryan, 2000; Martela & Ryan, 2020; Martela & Sheldon, 2019; Vansteenkiste et al., 2023), at least five key criteria can be offered that must be satisfied for something to be considered a psychological need.

*First*, the satisfaction of the psychological need should be directly connected to positive affective consequences. Psychological needs are seen as nutrients an organism needs to thrive and do well (Ryan & Deci, 2017). Accordingly, the satisfaction of a basic need should be rewarding in the sense of leading to increased positive feelings and improvements in other affective and evaluative indicators of well-being. Satisfaction of a psychological need should thus directly enhance well-being. As Ryan and Deci (2004) argued that “to qualify as a need, a motivating force must have a direct relation to well-being” (p. 22). *Second*, the frustration of the psychological need should be directly connected to negative affective consequences. Given the integral role of psychological needs in human wellness, their direct frustration should be consistently and directly associated with indicators of ill-being, such as negative affect and depressive symptoms (Vansteenkiste & Ryan, 2013). *Third*, the chronic satisfaction of the psychological need should lead to long-term functional benefits. Humans have certain psychological needs because they have oriented us toward contexts, situations, and behaviors that were “entailed in thriving during our species’ history” (Ryan & Deci, 2017, p. 84). Accordingly, beyond immediate affective consequences, the satisfaction of a psychological need should thus also lead to long-term functional consequences in terms of mental health, thriving, adaptive behavior, and good outcomes in various life contexts. For example, orienting toward activities one experiences high levels of competence in is arguably beneficial for success at work and other contexts, high sense of relatedness can prove crucial when one needs the help of friends, and autonomy-supportive contexts give more room to make choices optimal for one’s life outcomes (Ryan & Deci, 2017). The needs thus function analogously to Sen’s (1999) view of how capabilities relate to wellness. Conversely, growing up or living in conditions in which one or more of these basic needs is chronically deprived results in ill-being and developmental and social dysfunction (Vansteenkiste & Ryan, 2013). There thus should be “functional costs of need frustration or neglect and benefits of flourishing for satisfying them” (Ryan & Deci, 2017, p. 85). *Fourth*, the need should explain the well-being benefits of many behavioral and environmental factors. Psychological needs, by describing broad but specific experiences one can get from one’s interaction with the environment, should typically provide an explanation for why certain activities and certain environmental conditions lead to well-being. For example, increasing access to transportation in aging populations may increase well-being via its enhancement of autonomy (Schüz et al., 2016). Empirically, this means that the needs should mediate the link between various environmental factors and experiential indicators of well-being. *Fifth*, the need should be universally operational across cultural contexts and developmental periods. In being connected to human nature, any psychological need should be universal: It should have roughly equal effects around the world across all cultural contexts (Ryan & Deci, 2017, p. 85). Accordingly, for the four criteria listed above, one should be able to find robust cross-cultural evidence that the criteria are not only satisfied within one population but also across a wide range of cultural contexts from modern metropolises to hunter-gatherer societies.

As regards autonomy, competence, and relatedness as potential psychological needs, a long line of research has shown that they indeed are key predictors of experiential and evaluative well-being (reviewed in Ryan & Deci, 2017; Van den Broeck et al., 2016; Vansteenkiste et al., 2020), demonstrated by daily diary studies (e.g., Martela & Ryan, 2016; Reis et al., 2000; Ryan et al., 2010), longitudinal studies (e.g., Garn et al., 2019; Sheldon & Elliot, 1999; Tian et al., 2014), and experimental studies (e.g., Sheldon et al., 2010; Sheldon & Filak, 2008). Need frustration, in turn, has been shown to relate to depression, negative affect, and burnout (Bartholomew et al., 2011), stress (Campbell et al., 2017), and depressive symptoms (Chen, Vansteenkiste, et al., 2015). A recent systematic review (Ryan et al., 2022) identified 56 meta-analyses on various dimensions of self-determination theory, including 12 focusing on basic psychological needs, such as the relation of need satisfaction with positive and negative affect (Stanley et al., 2021), well-being at work (Van den Broeck et al., 2016), well-being among elderly people (Tang et al., 2020), burnout among athletes (Li et al., 2013), and performance (Cerasoli et al., 2016), in general providing consistent support for the theory. The main results on well-being effects have also been shown to be robust in cross-cultural research (Chen, Vansteenkiste, et al., 2015; Church et al., 2013; Sheldon et al., 2001; Martela et al. 2022) as well as in various populations such as adolescents (Jang et al., 2009) and elderly people (Kasser & Ryan, 1999) and in contexts ranging from education (e.g., Jang et al., 2016) and sports coaching (e.g., Curran et al., 2016) to work (Van den Broeck et al., 2016). For example, a study utilizing Gallup World Poll data from 155 countries showed that indicators for the three needs predicted evaluative and experiential well-being quite equally across the world regions (Tay & Diener, 2011), and a meta-analysis of 36 samples showed no difference in the size of correlation between autonomy and indicators of evaluative and experiential well-being in the United States and East Asia (Yu et al., 2018).

Furthermore, the needs are seen to mediate the relationship between various environmental factors and evaluative and experiential well-being, as shown in research from diverse cultures. For example, longitudinal three-wave mediation studies have found that need satisfaction fully mediates the relations between (a) materialism and both life satisfaction and depression (Wang et al., 2017), (b) supportive teaching style and engagement in high school (Jang et al., 2016), (c) coach motivational style and engagement in youth sports (Curran et al., 2016), and (d) self-critical perfectionism and binge-eating symptoms (Boone et al., 2014). Research has also shown that these three needs mediate between economic conditions (e.g., Di Domenico & Fournier, 2014), Rawl’s (2001) perceived primary goods (Bradshaw et al., 2021), Nussbaum’s (2000) perceived capabilities (DeHaan et al., 2016), and wellness outcomes. The needs for autonomy, competence, and relatedness thus seem to function as key experiences we need from our environment, the acquiring of which tends to lead to well-being and the deprivation of which tends to lead to ill-being.

There are several validated scales available to measure the satisfaction of the needs for autonomy, competence, and relatedness (e.g., Chen, Vansteenkiste, et al., 2015; Ilardi et al., 1993; Sheldon & Hilpert, 2012). The most popular scale, the Basic Psychological Need Satisfaction and Frustration Scale (BPNSFS; Chen, Vansteenkiste, et al., 2015), was initially validated in four languages and has now been translated into 15 languages. It includes four items for the measurement of each need, and given its good psychometric properties and wide usage, we recommend its usage for measuring need satisfaction. In situations in which there is room for only one item per need, we recommend the following items, based on our work to validate best-performing single items for each of the need (Martela & Ryan, 2022):

1. I am able to do things that I really want and value in life. (Autonomy)
2. I can do things well and achieve my goals. (Competence)
3. I feel close and connected with other people who are important to me. (Relatedness)

Although we have focused on the three psychological needs as recognized by SDT, our proposal on the third category of well-being being about psychological functioning is not dependent on accepting this particular list of needs. What we propose, in essence, is that the category of psychological functioning should be kept open but guided by clear criteria (Vansteenkiste et al., 2020). Autonomy, competence, and relatedness represent the empirically best supported current candidates for psychological needs and hence psychological functioning, but we must remain open to other potential candidates as well. Research within SDT, for example, has also actively examined other candidate needs such as beneficence (Martela & Ryan, 2016, 2020) and safety (Chen, Van Assche, et al., 2015; Rasskazova et al., 2016) to examine how well they fulfill the empirical criteria for a psychological need. And although SDT has focused on relatedness, other theorists have proposed somewhat overlapping needs related to human sociality, such as the need to belong (Baumeister & Leary, 1995) or the need for affiliation (McClelland, 1985). Thus, our main proposal is that psychological needs should be seen as a key category in broader assessments of well-being wanting to tap into psychological functioning because this would provide clear empirical criteria for what counts as psychological functioning, leading to a parsimonious set of elements to be measured that are universally important for the short-term and long-term wellness and thriving of humans.

## The Role of Meaning in Life in Broader Assessments of Well-Being

Although the focus of the present article is on psychological needs as one key part of broader assessments of well-being, it is worth discussing briefly the role of meaning in such assessments, given that meaning and purpose in life has been the other prominent way of defining and operationalizing the third, eudaimonic category of well-being.

In our view, meaning in life might be best understood not as a third category of well-being beyond evaluative and experiential well-being but rather as a separate type of evaluative well-being beyond life satisfaction. Evaluative well-being, as noted, is the cognitive component of well-being, focusing on a reflective assessment of a person’s life as a whole (Diener et al., 1999; OECD, 2013). It is typically covered by questions examining the satisfaction with one’s life as a whole. However, judgments about the meaning and purpose of one’s life as a whole seem to be similar to judgments about life satisfaction in being cognitive overall assessments of the quality of one’s life. Steptoe et al. (2015), for example, observed that as regards the “cognitive processing necessary,” feelings can be reported fairly directly, “whereas life evaluations and meaning questions are likely to demand substantial thinking, including aggregation over time and comparison with self-selected standards” (p. 641). Although evaluative well-being has been typically covered solely by an evaluation of life satisfaction, we suggest that complementing this with an evaluation of the meaningfulness and purpose of one’s life would ensue a richer view of how the person cognitively evaluates their life: Are they satisfied with it, and do they find meaning and purpose in it? Both can be seen as fundamental evaluations of one’s life as a whole and should thus be seen as two separate types of evaluative well-being. However, because this is a conceptual proposal, we invite empirical research to more carefully examine the relation between life satisfaction and meaning in life to better understand how the two types of cognitive judgments about life complement each other.

As regards the relation between purpose/meaning and psychological needs, meaning in life has sometimes been treated as a need (Frankl, 1963). However, experimental research shows that it is often a consequence of experiential well-being rather than a predictor of it (King et al., 2006). Furthermore, psychological needs for autonomy and relatedness have been shown to be key predictors of meaning in life (Lambert et al., 2013; Martela et al., 2018, 2021; Schlegel et al., 2011). This means that meaning in life could serve as a useful proxy of the experiences that follow the satisfaction of psychological needs. Accordingly, Ryan and Deci (2017) observed that “meaning is better viewed as an outcome of basic need satisfaction than as a basic need in its own right” (p. 254). This further suggests that meaning in life could serve as a type of evaluative well-being sensitive to levels of psychological functioning, whereas the elements of psychological functioning are the types of experiences that consistently lead to positive life evaluation, such as a sense of meaning in life.

## Discussion

The direct measurement of subjective well-being at a national level has the potential to change the politics to better serve the key goal of public policymaking: improving the well-being of the citizens. However, to realize that goal, we need reliable, comparative, and cross-culturally tested indicators of subjective sense of well-being. The important developments in measuring evaluative well-being and experiential well-being have led to widely accepted best practices (Diener et al., 2018; Graham et al., 2018; OECD, 2013), and standardized measurements across countries have made possible a science of national well-being in which various political and cultural factors are examined as potential predictors of national differences in well-being (e.g., Helliwell, Huang, Grover, & Wang, 2018; Mikucka et al., 2017). This has led to research that is directly relevant for policymakers (Diener & Seligman, 2018) because it examines how factors such as quality of governance and democratic institutions (Helliwell & Huang, 2008; Ott, 2011), income inequality (Reyes-García et al., 2019; Schneider, 2016), gender inequality (Audette et al., 2019), and social capital and trust (Delhey & Dragolov, 2016; Helliwell, Huang, & Wang, 2018) affect national levels of well-being, and the development of a subjective sense of well-being within the nation has been shown to have a prominent effect on how people vote (Ward, 2020; Ward et al., 2021). However, what dimensions of well-being are visible in such policy-relevant research is restricted by what indicators are included in national accounts of well-being.

Cross-national, comparative, and policy-relevant research around the third dimension of well-being has been hindered by a lack of consensus around its definition and constitutive elements. To remedy this situation, we have proposed here that a prime category to be included in broader assessments of well-being is the satisfaction of human psychological needs that provide a way of capturing human psychological functioning and well-doing. Measuring basic psychological needs could provide a parsimonious set of indicators for the third dimension of well-being, focusing on key experiences humans across the world need to thrive and do well in life. On the basis of research on SDT, a strong case can be built around recognizing autonomy, competence, and relatedness as such universally required psychological needs, although the exact list of needs ought to be left to be updated on the basis of cumulative empirical evidence.

Various measures for psychological needs have already been used in cross-cultural research such as the Gallup World Poll, with research demonstrating the universality and substantial independence of the effects of the needs on evaluative and experiential well-being (Tay & Diener, 2011) and how the psychological needs operate as mediators between material prosperity and evaluative/experiential well-being (Diener et al., 2010). The personal and social well-being module of the European Social Survey also includes measures for the three psychological needs of SDT (Conzo et al., 2017;Huppert et al., 2009; Martela et al. 2022). Most recently, the Citizen Pulse Survey conducted in Finland included (in consultancy with the first author) measures for autonomy, competence, and relatedness in their nationally representative monthly survey. Given that there are well-validated surveys translated in several languages for measuring the psychological needs (Chen, Vansteenkiste, et al., 2015) and proposals about how to measure the needs when there is room for only one item per need (see above), we encourage and anticipate more national-level and cross-cultural research studies adopting measures of need satisfaction in their surveys. The inclusion of standardized measures for the satisfaction of psychological needs is important to make possible international comparisons and cross-national research (e.g., see Conzo et al., 2017).

The practical advantage of measuring psychological needs alongside experiential and evaluative well-being is that it defines more specific content compared with the essentially content-free measures of life satisfaction and affect (Martela & Sheldon, 2019). Knowing that life satisfaction is decreasing is not alone very helpful for policymakers. But knowing that it is accompanied by (and explained by) a similar decrease in relatedness already reveals much more about the problem and how to start to resolve it. For example, during the COVID-19 pandemic a large number of employees were suddenly forced into a long period of remote work. Although this arguably could even have a positive effect on work autonomy, the loss of relatedness seemed to lead to a decrease in work engagement and increase in burnout (Kaltiainen & Hakanen, 2022). Indicators of need satisfaction would thus go beyond mere observations that subjective well-being is increasing or decreasing by starting to explain why this is happening, thus pointing toward an explanation. Measuring psychological needs would provide a fuller picture of how the citizens are doing and what could help them do even better in the future.

Although our proposal focuses on the inclusion of indicators for needs for autonomy, competence, and relatedness in national accounts of well-being, we welcome other proposals for what else should be included in broader assessments of well-being. For example, it is important to carry out more research on other candidate needs to identify whether there are other psychological needs in addition to the three needs established by SDT. Furthermore, both psychological functioning and eudaimonia are broad constructs; thus, there are many other important psychological constructs that could arguably be part of broader assessments of well-being ranging from meaning in life and optimism to sense of security and prosociality (Huppert & So, 2013; Keyes, 2007; VanderWeele et al., 2020). Each of the constructs would require a separate assessment of the merits and arguments for their inclusion. We hope that by providing the argument for why psychological needs should be included in national accounts of well-being we inspire other researchers to provide the arguments for other candidates, thus leading to a fruitful dialogue through which a consensus around the key elements of national accounts of well-being can be identified.

In summary, we propose that standardized indicators of psychological needs should be measured alongside indicators of evaluative and experiential well-being in national and international surveys. Their inclusion would give policymakers a parsimonious set of indicators on crucially important dimensions of well-doing—thus enriching their understanding of the wellness of the citizens. Psychological needs would also provide central mediator variables for explaining how various cultural, economic, and social factors concretely affect citizens’ well-being and health. Focusing future examinations of the third dimension of well-being on psychological functioning and psychological needs would provide theoretical unity, construct clarity, and international comparability and a way of measuring well-being on a national level that is more useful for the policymakers and thus more impactful in advancing the well-being of the citizens.

## Transparency

*Action Editor:* Klaus Fiedler

*Editor:* Klaus Fiedler
